# Supplementary material for: Dissecting the binding mechanisms of transcription factors to DNA using a statistical thermodynamics framework
Source: Comput Struct Biotechnol J. 2020 Nov 12;18:3590–605. doi: 10.1016/j.csbj.2020.11.006 (PMC7708957; doi:10.1016/j.csbj.2020.11.006)
Supplement: Supplementary file 2 [file mmc2.pdf]

Supplementary Tables to:
Dissecting the binding mechanisms of transcription factors to DNA using a statistical thermodynamics framework.

Table S1: Data sources for all TFs. Data sets have been separated into modEncode ChIP data [1] , GEO ChIP data, Accessibility Data, and ENCODE data for *Homo sapiens* [1, 2]

Cell Line	TF	modEncode Accession #	Type
S2	CTCF	2638	WT
S2	CTCF	2639	WT
S2	CTCF	283	WT
S2	CTCF	3281	WT
S2	CTCF	3749	WT
S2	CTCF	913	WT
S2	BEAF-32	922	WT
S2	BEAF-32	3745	WT
S2	BEAF-32	274	WT
S2	Su(Hw)	3719	WT
S2	Su(Hw)	330	WT
S2	Su(Hw)	331	WT
BG3	CTCF	282	WT
BG3	CTCF	3280	WT
BG3	BEAF-32	3663	RNAi
BG3	BEAF-32	3664	RNAi
BG3	BEAF-32	3665	WT
BG3	BEAF-32	921	WT
BG3	CTCF	3671	RNAi
BG3	CTCF	3672	WT
BG3	CTCF	3673	WT
BG3	CTCF	3674	RNAi
BG3	Su(Hw)	3714	RNAi
BG3	Su(Hw)	3715	RNAi
BG3	Su(Hw)	3716	WT
BG3	Su(Hw)	3717	RNAi
BG3	Su(Hw)	3718	WT
BG3	Su(Hw)	951	WT
Kc167	CTCF	908	WT
Kc167	Su(Hw)	3801	WT

Cell Line	TF	GEO Accession #
Kc167	CTCF	GSE30740
Kc167	Su(Hw)	GSE30740
Kc167	BEAF-32	GSE30740
Kc167	BEAF-32	GSE62904

Cell Line	Method	GEO Accession #
Kc167	DNase	Kharchenko et al. 2011
S2	DNase	Kharchenko et al. 2011
BG3	DNase	Kharchenko et al. 2011
Kc167	ATAC-seq	GSE122575

Cell Line	Method	File Type	ENCODE #
Astrocyte	DNase	BAM	ENCFF384CCQ
Astrocyte	DNase	BAM	ENCFF885IAD
Astrocyte	DNase	BigWig	ENCFF901UBX
Astrocyte	DNase	bed	ENCFF021SAS
Astrocyte	DNase	bed	ENCFF352LYZ
Astrocyte	ChIP-seq	bigWig	ENCFF424JNY
Astrocyte	ChIP-seq	bed	ENCFF600CYD
Astrocyte	ChIP-seq	bed	ENCFF183YLB

Table S2: Goodness of Fit metrics: ChIPanalyser offers 12 goodness of fit metrics grouped into two classes *Dissimilarity* and *Similarity*. Symbolically each metric is either a measure of how different two datasets are (Dissimilarity) or a measure of how similar two datasets are (Similarity). TP - true positives, TN - true negatives, FP - false positives and FN - false negatives. MCC represents Matthews Correlation Coefficient.

Metric	Description	Type
MSE	Mean Squared Error	Dissimilarity
K-S distance	Kolmogorov-Smirnov Goodness-of-Fit Test	Dissimilarity
Geometric ratio	$\frac{\int_a^b \lvert f(x)-g(x) \rvert dx}{\int_a^b min(f(x),g(x))dx}$	Dissimilarity
Recall	$\frac{TP}{TP+FN}$	Dissimilarity
Pearson	Pearson correlation coefficient between predicted and ChIP profiles	Similarity
Spearman	Spearman correlation coefficient between predicted and ChIP profiles	Similarity
Kendall	Kendall correlation coefficient between predicted and ChIP profiles	Similarity
Precision	$\frac{TP}{TP+FP}$	Similarity
F-score	$\frac{2TP}{2TP+FP+FN}$	Similarity
Accuracy	$\frac{TP+TN}{TP+TN+FP+FN}$	Similarity
MCC	$\frac{TP\times TN-FP\times FN}{\sqrt{(TP+FP)(TP+FN)(TN+FP)(TN+FN)}}$	Similarity
AUC	Area Under the ROC (Receiver Operator Characteristic) Curve	Similarity

Table S3: Optimal set of parameters after training on top ten regions and minimising MSE From left to right, the white column shows the optimal parameters selected and associated MSE during training when No Accessibility was considered. Light grey represents optimal parameters selected and associated MSE during training when Continuous DNA accessibility scores were considered. Finally, dark grey are the optimal parameters selected and associated MSE when only DNase hypersensitivity sites (DHS) were considered.

TF	# Bound	Lambda	MSE	# Bound	Lambda	MSE	# Bound	Lambda	MSE
BG3 modEncode 282 CTCF	1e+06	0.75	0.009	1e+06	0.5	0.009	5e+05	0.75	0.009
BG3 modEncode 3280 CTCF	5e+05	0.75	0.013	5e+05	0.5	0.013	20000	1.5	0.018
BG3 modEncode 3671 CTCF	1e+06	0.75	0.008	1e+06	0.5	0.008	5e+05	0.75	0.007
BG3 modEncode 3672 CTCF	1e+06	0.5	0.006	50000	0.5	0.006	1e+05	1	0.007
BG3 modEncode 3673 CTCF	5e+05	0.75	0.01	20000	0.75	0.01	5e+05	0.75	0.008
BG3 modEncode 3674 CTCF	1e+06	0.75	0.01	1e+06	0.5	0.01	5e+05	0.75	0.011
Kc167 GSM762842 CTCF	50000	1	0.007	500	1	0.007	10000	1.5	0.006
Kc167 modEncode 908 CTCF	5e+05	0.75	0.011	50000	0.5	0.01	5e+05	0.75	0.009
S2 modEncode 2638 CTCF	50000	1.25	0.002	500	1.25	0.002	5000	1.5	0.002
S2 modEncode 2639 CTCF	20000	1.25	0.002	200	1.25	0.002	5000	2	0.002
S2 modENCODE 283 CTCF	5e+05	0.75	0.014	20000	0.5	0.013	1e+06	0.75	0.013
S2 modEncode 3281 CTCF	5e+05	0.75	0.017	20000	0.5	0.017	50000	2	0.019
S2 modEncode 3749 CTCF	1e+05	1	0.009	1000	1	0.009	20000	1.25	0.01
S2 modEncode 913 CTCF	2e+05	0.75	0.01	1e+06	0.25	0.01	5e+05	0.5	0.014
BG3 modEncode 3714 Su(Hw)	1e+05	2	0.023	5000	2	0.023	5000	2	0.045
BG3 modEncode 3715 Su(Hw)	1e+05	2	0.024	5000	2	0.024	10000	2.75	0.046
BG3 modEncode 3716 Su(Hw)	2e+05	1.5	0.016	5000	1.25	0.016	10000	2	0.031
BG3 modEncode 3717 Su(Hw)	1e+05	2	0.016	2000	1.75	0.016	10000	2.25	0.032
BG3 modEncode 3718 Su(Hw)	50000	1.75	0.012	1000	1.75	0.012	5e+05	1	0.019
BG3 modEncode 951 Su(Hw)	1e+05	1.75	0.017	2000	1.5	0.017	2e+05	0.75	0.041
Kc167 modEncode 3801 Su(Hw)	1e+05	2.25	0.017	5000	2.25	0.017	2e+05	1.25	0.03
Kc167 Su(Hw)	50000	1.25	0.004	200	1.25	0.004	10000	1.25	0.005
S2 modEncode 330 Su(Hw)	2e+05	1.75	0.022	5000	1.75	0.022	5e+05	0.75	0.05
S2 modEncode 331 Su(Hw)	1e+06	1.25	0.017	5000	1.25	0.017	5e+05	0.75	0.037
S2 modEncode 3719 Su(Hw)	2e+05	2.25	0.033	10000	2.25	0.032	2e+05	0.75	0.066
BG3 modEncode 3663 BEAF-32	1e+05	2.75	0.026	10000	2.5	0.026	50000	3	0.023
BG3 modEncode 3664 BEAF-32	1e+05	2.5	0.027	10000	2.5	0.027	1e+05	4	0.019
BG3 modEncode 3665 BEAF-32	2e+05	3	0.039	20000	2.75	0.04	2e+05	2.5	0.034
BG3 modEncode 921 BEAF-32	20000	1.25	0.012	2000	1.75	0.012	20000	2.5	0.008
Kc167 GSM1535963 BEAF32	50000	0.75	0.012	1000	1	0.012	50000	0.75	0.008
Kc167 GSM762845 BEAF32	20000	1	0.006	1000	1.25	0.006	10000	1.25	0.005
S2 modEncode 3745 BEAF-32	50000	1.25	0.017	1000	1	0.017	50000	3.25	0.014
S2 modEncode 922 BEAF-32	50000	1.5	0.014	2000	1.5	0.014	50000	3	0.009

Table S4: Optimal set of parameters after training on top ten regions and maximising AUC From left to right, the white column shows the optimal parameters selected and associated AUC during training when No Accessibility was considered. Light grey represents optimal parameters selected and associated AUC during training when Continuous DNA accessibility scores were considered. Finally, dark grey are the optimal parameters selected and associated AUC when only DNase hypersensitivity sites (DHS) were considered.

TF	# Bound	Lambda	AUC	# Bound	Lambda	AUC	# Bound	Lambda	AUC
BG3 modEncode 282 CTCF	5e+05	0.25	0.944	20000	0.25	0.943	1	0.25	0.885
BG3 modEncode 3280 CTCF	20000	0.25	0.895	2000	0.25	0.895	5e+05	0.25	0.812
BG3 modEncode 3671 CTCF	2e+05	0.5	0.936	5e+05	0.25	0.937	10000	1	0.946
BG3 modEncode 3672 CTCF	10	1	0.922	1	1	0.922	2000	1.25	0.832
BG3 modEncode 3673 CTCF	20000	0.5	0.903	1000	0.5	0.903	20	1.25	0.853
BG3 modEncode 3674 CTCF	1e+06	0.25	0.929	5e+05	0.25	0.929	500	0.75	0.847
Kc167 GSM762842 CTCF	1e+06	0.5	0.954	10000	0.5	0.955	1e+06	0.5	0.887
Kc167 modEncode 908 CTCF	1e+05	0.25	0.952	50	0.25	0.952	1	1.25	0.963
S2 modEncode 2638 CTCF	50000	0.5	0.99	100	0.5	0.99	5000	0.5	0.963
S2 modEncode 2639 CTCF	100	2	0.915	1	2	0.916	2e+05	0.25	0.857
S2 modENCODE 283 CTCF	1e+06	0.25	0.926	200	0.25	0.926	10	1.25	0.886
S2 modEncode 3281 CTCF	1e+06	0.25	0.882	10000	0.25	0.882	50	1	0.864
S2 modEncode 3749 CTCF	50000	0.25	0.857	10	0.25	0.857	1e+06	0.25	0.848
S2 modEncode 913 CTCF	2000	0.75	0.938	1	0.75	0.938	2000	0.5	0.872
BG3 modEncode 3714 Su(Hw)	20000	0.75	0.928	100	0.75	0.928	1	0.25	0.458
BG3 modEncode 3715 Su(Hw)	50	0.75	0.927	1	0.75	0.927	1	0.25	0.606
BG3 modEncode 3716 Su(Hw)	10000	0.75	0.929	50	0.75	0.93	1	0.25	0.677
BG3 modEncode 3717 Su(Hw)	5000	0.75	0.927	20	0.75	0.927	10000	1.25	0.662
BG3 modEncode 3718 Su(Hw)	1	0.75	0.93	10	0.75	0.93	20	1.5	0.766
BG3 modEncode 951 Su(Hw)	2000	0.75	0.931	10	0.75	0.931	200	0.25	0.583
Kc167 modEncode 3801 Su(Hw)	50000	0.75	0.925	20	0.75	0.925	20000	0.75	0.548
Kc167 Su(Hw)	1e+06	0.75	0.962	500	0.75	0.962	20000	1.25	0.915
S2 modEncode 330 Su(Hw)	50000	0.5	0.931	50	0.5	0.931	1	0.25	0.565
S2 modEncode 331 Su(Hw)	1e+05	0.5	0.937	100	0.75	0.937	5000	0.25	0.643
S2 modEncode 3719 Su(Hw)	10000	1	0.917	50	1	0.916	1	0.25	0.43
BG3 modEncode 3663 BEAF-32	20000	0.25	0.66	1000	0.25	0.661	1	2.25	0.81
BG3 modEncode 3664 BEAF-32	5000	4	0.789	1000	4	0.789	100	3.5	0.859
BG3 modEncode 3665 BEAF-32	500	1.5	0.726	1	1.5	0.726	10000	0.75	0.816
BG3 modEncode 921 BEAF-32	5000	4.25	0.831	1000	4	0.831	1e+05	3	0.897
Kc167 GSM1535963 BEAF32	2e+05	0.25	0.847	5000	0.25	0.847	1000	3.75	0.963
Kc167 GSM762845 BEAF32	10000	4	0.937	1000	4	0.937	50	4.25	0.986
S2 modEncode 3745 BEAF-32	2e+05	0.25	0.857	1000	0.25	0.857	200	3.75	0.869
S2 modEncode 922 BEAF-32	10000	4	0.825	2000	4.25	0.826	1	3	0.928

Table S5: Optimal set of parameters after training on top ten regions and maximising recall From left to right, the white column shows the optimal parameters selected and associated recall during training when No Accessibility was considered. Light grey represents optimal parameters selected and associated recall during training when Continuous DNA accessibility scores were considered. Finally, dark grey are the optimal parameters selected and associated recall when only DNase hypersensitivity sites (DHS) were considered.

TF	# Bound	Lambda	Recall	# Bound	Lambda	Recall	# Bound	Lambda	Recall
BG3 modEncode 282 CTCF	20000	0.75	0.887	1000	0.75	0.887	1	1.5	0.778
BG3 modEncode 3280 CTCF	10000	0.75	0.816	500	0.75	0.818	1	1.25	0.676
BG3 modEncode 3671 CTCF	10000	0.75	0.884	500	0.75	0.884	1000	1.5	0.859
BG3 modEncode 3672 CTCF	1	1.25	0.883	1	1.25	0.883	5000	1.5	0.693
BG3 modEncode 3673 CTCF	20000	0.75	0.855	500	0.75	0.854	100	1.5	0.758
BG3 modEncode 3674 CTCF	10000	0.75	0.865	2000	0.75	0.865	100	1.5	0.735
Kc167 GSM762842 CTCF	5e+05	0.75	0.932	5000	0.75	0.932	10000	1.75	0.807
Kc167 modEncode 908 CTCF	10000	0.75	0.88	50	0.75	0.88	1	1.25	0.831
S2 modEncode 2638 CTCF	10000	1	0.962	100	1	0.962	2000	1.5	0.859
S2 modEncode 2639 CTCF	100	2	0.891	1	2.25	0.892	2000	1.75	0.727
S2 modENCODE 283 CTCF	10000	0.75	0.86	50	0.75	0.86	1	1.5	0.744
S2 modEncode 3281 CTCF	20000	0.75	0.805	50	0.75	0.805	500	1.25	0.71
S2 modEncode 3749 CTCF	20000	1	0.816	20	1	0.814	2000	1.25	0.682
S2 modEncode 913 CTCF	2000	1	0.875	20	1	0.875	1	1.75	0.711
BG3 modEncode 3714 Su(Hw)	500	1.25	0.85	20	1.25	0.85	10000	1.25	0.208
BG3 modEncode 3715 Su(Hw)	1000	1.25	0.848	10	1.25	0.848	10	2.25	0.397
BG3 modEncode 3716 Su(Hw)	20000	1.25	0.86	50	1.25	0.86	10000	1.5	0.443
BG3 modEncode 3717 Su(Hw)	2000	1.25	0.855	20	1.25	0.855	10000	1.25	0.419
BG3 modEncode 3718 Su(Hw)	5000	1.25	0.869	50	1.25	0.868	1	2	0.547
BG3 modEncode 951 Su(Hw)	5000	1.25	0.846	50	1.25	0.846	1	4.25	0.346
Kc167 modEncode 3801 Su(Hw)	10	1.25	0.851	1	1.25	0.851	20	1.5	0.225
Kc167 Su(Hw)	50000	1	0.936	50	1	0.935	20000	1.25	0.833
S2 modEncode 330 Su(Hw)	5000	1.25	0.841	20	1.25	0.841	5000	1.5	0.286
S2 modEncode 331 Su(Hw)	5000	1.25	0.863	20	1.25	0.862	10000	1.25	0.406
S2 modEncode 3719 Su(Hw)	5000	1.25	0.813	10	1.25	0.812	1e+05	1	0.132
BG3 modEncode 3663 BEAF-32	10	1	0.627	20	1	0.627	1	2.75	0.69
BG3 modEncode 3664 BEAF-32	5000	4	0.762	1000	4	0.761	5000	4	0.732
BG3 modEncode 3665 BEAF-32	2000	2	0.687	200	2	0.687	2e+05	1.75	0.663
BG3 modEncode 921 BEAF-32	5000	4.25	0.807	1000	4.25	0.806	1e+05	3	0.829
Kc167 GSM1535963 BEAF32	1	1.5	0.823	1	1.5	0.823	1000	3.75	0.902
Kc167 GSM762845 BEAF32	10000	4	0.918	1000	4	0.918	50	4.25	0.95
S2 modEncode 3745 BEAF-32	1000	0.75	0.806	20	0.75	0.806	1	3.25	0.745
S2 modEncode 922 BEAF-32	10000	4	0.799	2000	4.25	0.801	2000	3.25	0.856

Table S6: Optimal set of parameters after training on top ten regions and maximising Spearman correlation From left to right, the white column shows the optimal parameters selected and associated spearman correlation during training when No Accessibility was considered. Light grey represents optimal parameters selected and associated Spearman correlation during training when Continuous DNA accessibility scores were considered. Finally, dark grey are the optimal parameters selected and associated Spearman correlation when only DNase hypersensitivity sites (DHS) were considered.

TF	# Bound	Lambda	spearman	# Bound	Lambda	spearman	# Bound	Lambda	spearman
BG3 modEncode 282 CTCF	1e+06	0.25	0.584	2e+05	0.25	0.584	5e+05	0.25	0.523
BG3 modEncode 3280 CTCF	1e+06	0.25	0.512	2e+05	0.25	0.513	5e+05	0.25	0.461
BG3 modEncode 3671 CTCF	1e+06	0.25	0.582	1e+06	0.25	0.583	1	0.25	0.581
BG3 modEncode 3672 CTCF	1e+05	0.5	0.502	5000	0.5	0.503	1	0.25	0.404
BG3 modEncode 3673 CTCF	1e+06	0.25	0.463	1e+06	0.25	0.464	1	0.25	0.462
BG3 modEncode 3674 CTCF	1e+06	0.25	0.483	50000	0.25	0.483	2000	0.5	0.471
Kc167 GSM762842 CTCF	1	0.25	0.299	5e+05	0.25	0.312	20	3.25	0.367
Kc167 modEncode 908 CTCF	5e+05	0.25	0.551	100	0.25	0.551	10	1.25	0.638
S2 modEncode 2638 CTCF	1e+06	0.25	0.608	10000	0.25	0.608	5e+05	0.25	0.544
S2 modEncode 2639 CTCF	1	0.25	0.438	10000	0.25	0.438	1	0.25	0.323
S2 modENCODE 283 CTCF	1	0.25	0.596	20000	0.25	0.597	1	0.25	0.517
S2 modEncode 3281 CTCF	1e+06	0.25	0.496	500	0.25	0.496	5e+05	0.25	0.448
S2 modEncode 3749 CTCF	1e+06	0.25	0.446	10000	0.25	0.448	5e+05	0.25	0.467
S2 modEncode 913 CTCF	5e+05	0.25	0.613	10000	0.25	0.614	200	1.25	0.556
BG3 modEncode 3714 Su(Hw)	5e+05	0.5	0.689	1000	0.5	0.689	1	0.25	-0.109
BG3 modEncode 3715 Su(Hw)	50	0.75	0.704	1	0.75	0.704	1	0.25	0.1
BG3 modEncode 3716 Su(Hw)	1000	0.75	0.705	1	0.75	0.705	1	0.25	0.22
BG3 modEncode 3717 Su(Hw)	2000	0.75	0.682	1	0.75	0.682	5e+05	1	0.238
BG3 modEncode 3718 Su(Hw)	1	0.5	0.647	50	0.5	0.645	1	0.75	0.462
BG3 modEncode 951 Su(Hw)	5e+05	0.5	0.718	20000	0.5	0.721	1	0.25	0.056
Kc167 modEncode 3801 Su(Hw)	1e+06	0.5	0.674	200	0.5	0.674	1e+05	0.75	0.062
Kc167 Su(Hw)	50000	0.75	0.421	20	0.75	0.428	200	1.5	0.361
S2 modEncode 330 Su(Hw)	1e+06	0.5	0.748	1e+06	0.25	0.757	1	0.25	0.037
S2 modEncode 331 Su(Hw)	5e+05	0.5	0.675	1e+06	0.25	0.691	1	0.25	0.194
S2 modEncode 3719 Su(Hw)	50000	0.75	0.743	100	0.75	0.746	1	0.25	-0.266
BG3 modEncode 3663 BEAF-32	100	0.5	0.16	1	0.5	0.16	500	0.5	0.503
BG3 modEncode 3664 BEAF-32	500	0.25	0.317	20	0.25	0.317	1000	1.75	0.507
BG3 modEncode 3665 BEAF-32	50000	0.25	0.341	1	0.75	0.34	50000	0.75	0.512
BG3 modEncode 921 BEAF-32	1e+06	0.25	0.252	1e+05	0.25	0.253	1000	0.5	0.31
Kc167 GSM1535963 BEAF32	2e+05	0.25	0.385	2000	0.25	0.385	1000	3	0.575
Kc167 GSM762845 BEAF32	1e+06	0.25	0.297	10000	0.25	0.296	200	4	0.552
S2 modEncode 3745 BEAF-32	50000	0.25	0.385	200	0.25	0.385	50	2.25	0.517
S2 modEncode 922 BEAF-32	5e+05	0.25	0.238	5000	0.25	0.237	5000	1.75	0.448

References

[1] Davis, C.A., Hitz, B.C., Sloan, C.A., Chan, E.T., Davidson, J.M., Gabdank, I., Hilton, J.A., Jain, K., Baymuradov, U.K., Narayanan, A.K., Onate, K.C., Graham, K., Miyasato, S.R., Dreszer, T.R., Strattan, J., Jolanki, O., Tanaka, F.Y., Cherry, J.: The Encyclopedia of DNA elements (ENCODE): data portal update. Nucleic Acids Res. **46**(D1), 794–801 (2018). doi:10.1093/nar/gkx1081

[2] ENCODE Project Consortium: An integrated encyclopedia of DNA elements in the human genome. Nature **489**(7414), 57–74 (2012). doi:10.1038/nature11247
